# DeepLumina: A Method Based on Deep Features and Luminance Information for Color Texture Classification

**DOI:** 10.1155/2022/9510987

**Published:** 2022-04-14

**Authors:** A. Philomina Simon, B. V. Uma

**Affiliations:** ^1^Department of Computer Science, University of Kerala, Thiruvananthapuram, Kerala, India; ^2^Department of Computer Science, Pondicherry Central University, Puducherry, India

## Abstract

Color texture classification is a significant computer vision task to identify and categorize textures that we often observe in natural visual scenes in the real world. Without color and texture, it remains a tedious task to identify and recognize objects in nature. Deep architectures proved to be a better method for recognizing the challenging patterns from texture images. This paper proposes a method, DeepLumina, that uses features from the deep architectures and luminance information with RGB color space for efficient color texture classification. This technique captures convolutional neural network features from the ResNet101 pretrained models and uses luminance information from the luminance (Y) channel of the YIQ color model and performs classification with a support vector machine (SVM). This approach works in the RGB-luminance color domain, exploring the effectiveness of applying luminance information along with the RGB color space. Experimental investigation and analysis during the study show that the proposed method, DeepLumina, got an accuracy of 90.15% for the Flickr Material Dataset (FMD) and 73.63% for the Describable Textures dataset (DTD), which is highly promising. Comparative analysis with other color spaces and pretrained CNN-FC models are also conducted, which throws light into the significance of the work. The method also proved the computational simplicity and obtained results in lesser computation time.

## 1. Introduction

The texture represents the surfaces based on roughness, regularity, repeating patterns, homogeneity, and granularity [1]. Textured surfaces in the real world can be categorized based on their homogeneity or heterogeneity, coarse or grain details, rough or smooth patterns, regularity or irregularity, and structural or nonstructural patterns within an image. Identifying and classifying texture patterns across nature are essential in machine vision applications such as automated visual inspection, visual fine-grained object classification, forest species classification, wood classification, and fabric defect classification. Materka et al. [2] discussed texture classification methods, which include statistical, structural, model-based, and transform-based methods. Texture features and color are vital visual attributes for identifying the realistic view of the natural scenes such as grass, tree bark, flowers, food grains, wood, fabric, and metals through color texture classification. The texture-color amalgamation is a perfect model, and it is effective in classifying texture images [3]. Usually the color images are represented in RGB color space for pattern recognition and computer vision applications. The significance of the color spaces other than RGB for better color representations is not often investigated [4]. Since different color spaces have distinct color representation patterns, it affects the accuracy of the machine vision algorithm [5–7]. Kahu et al. [8] discussed the lack of luminance information in the RGB color space in image compression applications. Since RGB color space is not well suited for human perception, it has no direct luminance information [9]. RGB color space cannot capture luminance information during the processing of texture images. Kahu et al. inferred that RGB color space is not efficient for processing the images from natural, realistic scenarios and also for machine vision applications such as emotion recognition and image classification [8]. This work throws light into the efficiency of the luminance information applied with RGB color space which can perceive the texture features better. Luminance represents the number of photons (light) emitted to pass through a textured surface. The human brain captures the visual attributes of texture and luminance from a natural scene [10]. In neurophysiology and visual psychophysics, we find that statistical characteristics of the visual scene are better perceived by humans who are based on the biologically inspired responses from the brain [11]. Johnson et al. [12] discussed that the biological responses correspond to the first-order information “luminance” and second-order response “texture” information in natural images. This work demonstrated the significance of luminance and texture information, the first-order and second-order information captured from the realistic natural images as filter response strength and a mutual correlation. Okada et al. [13] discussed that visual texture perception constitutes energy and luminance. In luminance, the visual texture features can be represented better. Deep convolutional neural network (CNN) behaves like the visual cortex brain. Deep CNN applies a kernel or filters of various sizes in a texture image to generate a feature map representing the image with its features.

With the significant increase in computation facilities, the deep learning framework continued to be a thriving research area for efficient texture classification. Literature demonstrates that representing texture based on deep neural networks is a promising thrust area to recognize natural textures. Last decade onwards, active progress in deep learning research grabbed attention in solving computationally intensive machine vision tasks. Deep learning models are better at perceiving the pattern or features from an image. The major challenge of deep learning for classification tasks is that it requires enormous computation time for processing an algorithm. The success of the convolutional neural networks design relies upon how well the hyperparameters are chosen and fine-tuned. Despite the advancements in handcrafted features, convolutional neural network (CNN) models generate better-learned feature patterns corresponding to the input image. The deep network constructs feature with the edges, corners, contours, regions based on the feature layers from CNN pretrained models. In convolution operation, applying the same filter to input results obtain a map of activation values; they are feature maps [14]. Feature maps indicate the location, strength, orientation response of a detected feature in an input image. These feature maps better represent deep features and the luminance information in a texture image. The proposed method DeepLumina is designed on RGB and luminance-based color space in which luminance is generated from YIQ color space and deep features from pretrained ResNet101 model. Even though accuracy is an inevitable factor in assessing the algorithm performance, computational time also plays a significant part in affecting the performance and design of a vision based application.

The contributions of the proposed work are summarized as follows: In DeepLumina, we investigate the efficiency of applying deep features and luminance extracted from different color models and classify features using Support Vector Machine. DeepLumina uncovers the significance of luminance from YIQ color space and applies ResNet101 pretrained model for color texture classification. The accuracy of DeepLumina showed a substantial improvement in YIQ color space over the different color models when tested in the challenging texture datasets DTD and FMD. Computation time is minimal for DeepLumina.

The organization of the paper proceeds as follows.  Section 2 presents concise related literature works on deep neural networks for texture classification.  Section 3 elaborates the materials and methodology and explains the proposed approach DeepLumina for texture classification.  Section 4 discusses the experiments and analysis of the results obtained.  Section 5 describes a concise description of the contribution and compendium of the paper.

## 2. Related Research

Texture represents the statistical characteristics and the repetitive arrangement of patterns on a surface. Discriminating and understanding the texture patterns in an image is a significant task, especially in pattern recognition applications since the 1960s [15] and 1980s [16, 17]. It is still a tedious task to classify and represent the complex textures of objects that we encounter in our daily life. Color texture classification has been an active research area over the past four decades. In state-of-the-art techniques, the texture classification method selects appropriate handcrafted texture descriptors and classification is performed. The feature descriptors commonly used are Local Binary Pattern (LBP), LBP variants, Gray Level Co-occurrence Matrix (GLCM), and Law's Texture, which demonstrate better texture representations. The widely used classifiers are K-Nearest Neighbour with Chi-Squared Distance and Support Vector Machine [18].

**Figure 1:**
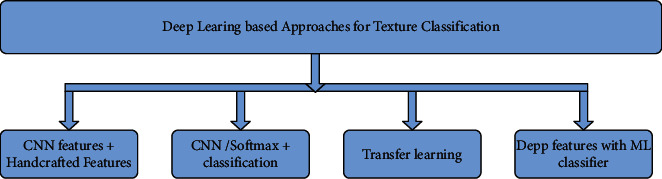
Deep Neural Network Approaches for color texture Classification.

Deep Texture Classification is a subclass of machine learning where the neural network automatically extracts the distinct image features which characterize the textures. The authors elaborated different approaches for deep texture classification and represented in Figure 1.

First approach focuses on the usage of Convolutional Neural Network (CNN) and handcrafted features for texture classification. Also, it exploits the possibility of applying the machine learning classifier along with deep CNN features. CNN can function as a feature extractor as well as a classifier. In this approach, the feature maps generated have better characteristics of the deep features and handcrafted features. Here CNN can perform feature extraction as well as classification. The second approach is to develop and design a new CNN architecture that extracts texture features and performs classification. It is necessary to ensure the selection of proper optimizers and hyperparameters for classification. The usage of the well-known pretrained models is another approach and such models perform transfer learning. This method uses the pretrained model where the model has thoroughly learned features from the vast ImageNet database. This approach is ideal when the number of images in the dataset is less and computational facilities are limited. Another is a blended approach that utilizes both machine learning classifier and deep learning feature generation techniques.

In DeepLumina, the deep features are extracted using pretrained ResNet101 model, which captures luminance information from YIQ color space and color information from RGB images. Texture classification is performed using a machine learning based classifier support vector machine (SVM). A brief review of approaches to deep texture classification is discussed in this section.

### 2.1. Hybrid CNN-Handcrafted Features with Machine Learning Classifier

Wang et al. [19] discussed deep neural network based feature fusion for identifying the malignancies in breast cancer. The authors used hybrid CNN and handcrafted features such as texture, density, morphological features, and an extreme machine learning classifier. Shi et al. [20] used the feature fusion for ship classification to get a MultiScale-Completed Local Binary pattern (MS-CLBP) and deep features and classified the image features using a support vector machine. These features capture the rotation invariance of the ship images. JBene et al. [21] discussed a method based on handcrafted and CNN features for texture classification. This work demonstrated that statistical features boost the classification performance of CNN when the deep features are concatenated with the handcrafted features—in that work, pretrained models, namely Xception and ResNet50 networks with handcrafted features LBP histogram, GLCM, Wavelet histogram, and Scale Invariant Feature transform-Fisher Vector (SIFT-FV) features for classification.

### 2.2. Texture Classification Based on Pretrained Models

Roy et al. [22] proposed TexFusionNet, fusion-based CNN, where the pretrained AlexNet and VGGNet last dense layers are fused. A fully connected layer is utilized to the class scores with categorical entropy as the loss function. Saleem et al. [23] discussed the deep learning architecture for plant disease detection and classification. Cimpoi et al. [24] examined the significance of fisher vector (FV) based feature space and proposed FV-CNN, FC-CNN for real-life describable texture recognition. The backbone of the FC-CNN and FV-CNN is the VGG model. The work also presents another feature, Fully Connected CNN (FC–CNN), which extracts the shape features of a region present in the image. FV-CNN captures local features from the regions selected and eliminates the global information. Inspired by the fact that feature reduction from the high dimensional texture features leads to the improvement in accuracy, Song et al. [25] modified the Cimpoi et al. work by introducing the feature reduction and developing a discriminative neural network to reduce the feature dimensions. This work [25] demonstrated the efficiency of the CNN features and evaluated them in DTD, KTIPS2a, and FMD datasets. Motivated by the fact that the deep features can represent better texture feature space, Simon et al. [18] proposed a deep framework that extracts deep features from pretrained models and used a support vector machine for texture pattern classification to classify the images. This method is computationally efficient as fine-tuning the CNN hyperparameters is not required. Hafemann et al. presented a transfer learning based texture classification. This work [26] uses CNN to obtain a new feature vector representation based on softmax classification and machine learning classifiers.

## 3. Materials and Methods

In color texture classification, deep neural networks can better capture color and luminance features. The proposed method DeepLumina investigates the advantage of using RGB and luminance information captured from real-world texture images. RGB color space is display-oriented, whereas human eyes are sensitive to luminance. So incorporating the luminance from the YIQ model have a prominent influence in accuracy for the texture classification.

### 3.1. Materials

**Figure 2:**
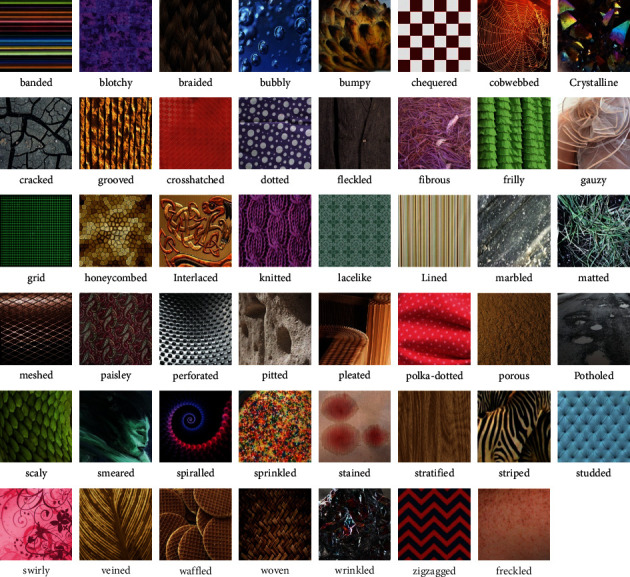
Glimpse of DTD dataset.

**Figure 3:**
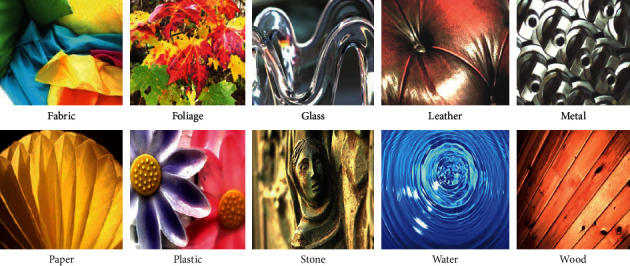
Glimpse of FMD dataset.

In color texture classification, the texture datasets Flickr Material Dataset (FMD) and Describable Texture Dataset (DTD) depict the various natural and describable textures in daily life. DTD and FMD provide a view of realistic textures that are commonly encountered in day-to-day life. These are complex datasets and are considered to be the most challenging for texture classification. Describable Textures dataset (DTD) is a colored-texture database [27] that contains the textures in the wild describing the real-world textures in nature such as cobwebbed, braided, dotted, blotchy, frilly. DTD has 120 images each for 47 texture categories adding up to 5640 images. The Flickr Material Database (FMD) [28] is another challenging texture dataset that captures the material appearance and visibility. FMD dataset has 10 classes, and each class contains 100 images. In the work, Liu et al. [3] considered DTD and FMD challenging because of large intraclass variations and appearance variations in the datasets. A glimpse of the FMD and DTD dataset is shown in Figures 2 and 3 respectively.

### 3.2. Significance of Color Spaces in Texture Classification

Color models [5] represent the images with different levels of perception, and it varies based on the discriminating power of the different colors and the intensity channels. Color spaces play a competent attribute for recognizing the visual scenes from real-world texture images. The selection of the color system has a significant influence on processing color images. The discriminative characteristics of a color space or color model vary based on the particular visual tasks and the provided texture image data. Cernadas et al. [29] categorized color systems as RGB based color space, visual perception based color models (HSV), and brightness-color based color spaces, which captures luminance chrominance (YCbCr, YUV, YIQ, L ^∗^ a ^∗^ b ^∗^) information. The limitation of the RGB color system is due to the high correlation between the *R*, G, B components [4]. Texture image or pattern can better be represented with RGB model with luminance obtained from color spaces including YIQ, YCbCr, HSV, and L ^∗^  a ^∗^ b ^∗^. In this work, the significance of luminance information from the above-mentioned color spaces is widely exploited for texture classification. Luminance information influences the representation of texture feature space. HSV and L ^∗^ a ^∗^ b [30] follow nonlinear transformation, whereas YCbCr and YIQ follow a linear transformation because of the visual perception of the images. The literature shows that HSV and YCbCr [31] color spaces are applied for face image retrieval applications and L ^∗^ u ^∗^ v ^∗^ and L ^∗^ a ^∗^ b ^∗^ in saliency detection. Broek et al. [32] presented a texture classification approach in applying texture descriptors such as color correlogram and cooccurrence matrix in six color spaces, including RGB, HSV, YUV, LUV, and YIQ. ColorNet is a CNN network developed by exploiting the concepts of the color spaces and pretrained models for classification. In ColorNet, Gowda et al. [6] used DenseNet model for classifying the CIFAR10 dataset and obtained good results.

### 3.3. Distinctiveness of Luminance

Luminance provides the illumination or brightness in an image. This channel extracts meaningful information by considering the illumination variance from a texture image [29]. L ^∗^ a ^∗^ b color space is similar to the HSV model in representing images based on human visual perception. According to Pietikainen et al. [33], distinct texture information can be found from the regions that represent high spatial frequency information from edges and boundaries. Prabhakar et al. [34] investigated that structure, edge details, and brightness variations can be extracted from the luminance channel than chrominance channels. Texture features can be effectively captured from luminance rather than color. Distinct color spaces provide diverse degrees of luminance separation from color [35]. *R*, *G*, and B variances are all similar for a given local area of space. Ware et al. [36] analyzed that the image feature information present in luminance channel and chroma channels are different. So it is advisable to segregate luminance from chroma dimensions in a color specification system. From the above literature, we can infer that the luminance channel is significant and there is a need to separate luminance from other color models for extracting the structural details and better feature representation for texture classification problems. In CNN-based superresolution [37], features are extracted from the luminance channel of YCbCr color space from deep neural networks. The significance of the luminance information needs to be better investigated for texture analysis applications. The literature shows that the color coordinate system can improve classification accuracy in different applications. The effect of luminance in color models with deep features needs further investigation. Li et al. [38] proved in their experiments that in deep network generated images, the chrominance components are more discernible than the luminance component. Deep generated images are more distinguishable in the residual domain of chrominance components. The deep network captures major chrominance features, and the image luminance information gets less prominence during the feature extraction. In such cases, the deep network may not capture the luminance information present in the image. It is worth promising to investigate the influence of luminance information for color texture classification. In our work, experimental analysis proved that texture classification accuracy improved incredibly when the deep network captured both color and luminance information. The impact of applying the luminance information from various color spaces and deep features influences the classification accuracy. The proposed method DeepLumina exploits the possibility of using luminance information with RGB color space, and it works in the RGB-Luminance feature space.

### 3.4. DeepLumina—Deep Learning Framework with Luminance Information for Color Texture Classification

In color texture classification, deep networks can better capture color and luminance features from RGB and luminance from the YIQ model, respectively. The proposed method investigated the impact of RGB and luminance information obtained from textures. In realistic natural images, first-order and second-order information corresponds to luminance and texture, respectively [12]. Inspired by the fact that luminance and texture are significant features that need to be captured from an image, we proposed DeepLumina.

**Figure 4:**
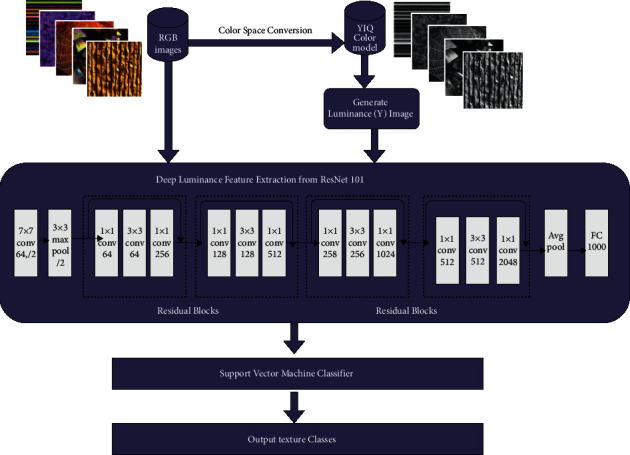
DeepLumina texture classification- proposed framework.

**Figure 5:**
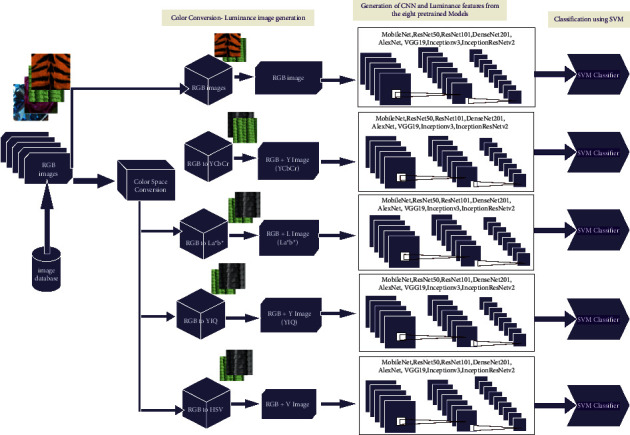
Framework of DeepLumina with color models and pretrained models.

The DeepLumina framework is summarized in Figure 4. Luminance provides better visual perception. The proposed method incorporated luminance along with RGB color features for deriving better texture feature space and performing classification by SVM. In the DeepLumina framework, we investigate the efficiency of luminance features with deep features. RGB texture images are converted to YCbCr, L ^∗^ a ^∗^ b ^∗^, YIQ, HSV color space and the corresponding luminance images are extracted. The chroma components are not considered since the color information can be preserved well in the RGB color space. These luminance images, along with the RGB images, are applied to the ResNet101 pretrained model. RGB images and Y channel from YIQ model (luminance) images are processed separately and then concatenated together into a feature vector for classification. The color features from RGB and luminance features are concatenated. The luminance channel has the power of discriminating the textures in a better way [34]. Pretrained models extract deep features and also capture luminance features from the texture images. These features are given to the fast linear solver support vector machine for classifying the real-world textures. RGB and the generated luminance images are trained using the ResNet101 model and it is compared with seven pretrained models for analysis. The DeepLumina framework with various color models and pretrained models is depicted in Figure 5. The procedure for the DeepLumina is presented in Table 1. Support vector machine (SVM) [39] can handle high dimensional texture feature space, so it can be efficiently applied for texture classification. The input images RGB are resized to 224 × 224 for applying in the ResNet101 model.

#### 3.4.1. YIQ Color Space Conversion and Luminance Image Estimation

Color systems have a detailed role to play especially in computer vision applications. In texture classification, different color space representations aid in improving accuracy [5]. RGB color space [40] represents the chromaticity of a particular color and no luminance channel is present in this color space. It is the most popularly used color model as it is device-oriented and prominently used in cameras display devices. RGB components are sensitive to lightness and shading. Texture images can be perceived better in the color spaces other than RGB as the RGB color system does not capture the aspects of the human visual system. RGB is nonuniform, whereas L ^∗^ a ^∗^ b ^∗^ is a perpetual uniform color space and HSV is an approximately uniform color space. Human perception can distinguish objects with hue, saturation, and intensity of colors as modeled in HSI space. Therefore, RGB color space is not effective in solving computer vision problems where performance is expected to match human vision [8]. So, it is necessary to investigate the color models other than the RGB model.

YIQ is also a nonlinear, nonuniform color space derived from RGB. *Y* represents Brightness/Luminance, *I* and *Q* represent the chroma or color components. The texture image in the YIQ model can be used for classification applications. In this work, we derive the *Y* component, and luminance is derived from the YIQ model for processing. The RGB to YIQ color conversion equation is given below [41]:

(1) $$\begin{array}{c} \left[\begin{array}{c} Y\\ I\\ Q \end{array}\right]=\left[\begin{array}{ccc} 0.299 & 0.587 & 0.114\\ 0.595 & -0.274 & -0.321\\ 0.211 & -0.522 & 0.311 \end{array}\right]\left[\begin{array}{c} R\\ G\\ B \end{array}\right]. \end{array}$$

L ^∗^ a ^∗^ b ∗ color system is based on the intermediate color space, namely CIE XYZ space. The RGB components are converted into the XYZ plane, then converted to L ^∗^ a ^∗^ b ^∗^ color space. In DeepLumina, we consider only the *L* channel of a textured image from L ^∗^ a ^∗^ b ^∗^ color space. RGB to L ^∗^ a ^∗^ b ^∗^ conversion is illustrated here [41, 42]:

(2) $$\begin{array}{c} R^{\prime }=\frac{R}{255}\\ G^{\prime }=\frac{G}{255}\\ B^{\prime }=\frac{B}{255}\\ \left[\begin{array}{c} X\\ Y\\ Z \end{array}\right]=\left[\begin{array}{ccc} 0.4124 & 0.3576 & 0.1805\\ 0.2126 & 0.7152 & 0.0722\\ 0.0193 & 0.1192 & 0.9505 \end{array}\right]\left[\begin{array}{c} R^{\prime }\\ G^{\prime }\\ B^{\prime } \end{array}\right],\\ \begin{array}{c} L^{*}=116\left[f\left(\frac{Y}{Y_{0}}\right)\right]-16\\ a^{*}=500\left[f\left(\frac{X}{X_{0}}\right)-f\left(\frac{Y}{Y_{0}}\right)\right]\\ b^{*}=200\left[f\left(\frac{Y}{Y_{0}}\right)-f\left(\frac{Z}{Z_{0}}\right)\right]\\ \text{where}f\left(x\right)=\left\{\begin{array}{cc} \frac{1}{x^{3}}, & \text{if }x>0.008856\\ 7.787x+\frac{16}{116}, & \text{otherwise} \end{array}\right. \end{array}. \end{array}$$

In L ^∗^ a ^∗^ b ^∗^ color space, colors are assigned with respect to a reference white point. *X*_0_, *Y*_0_, *Z*_0_ denote the coordinates of the reference white. L ^∗^ captures the luminance, a ∗ extracts the color variations from green to red and b ^∗^ represents the color variations from blue to yellow. In DeepLumina, the RGB texture images are converted to the YCbCr model, and only the Luminance image of the YCbCr is constructed for processing the algorithm. YCbCr color system is derived from the RGB model, and it is a perpetual nonlinear transformation of the RGB color model. YCbCr color space represents an image as Y, the luma component, and CbCr, the chroma components. YCbCr model is used in compression algorithms such as JPEG, JPEG 2000, H.264, HEVC.

The color conversion from RGB to YCbCr is equated as follows [43]:

(3) $$\begin{array}{c} \left[\begin{array}{c} Y\\ Cb\\ Cr \end{array}\right]=\left[\begin{array}{ccc} 65.48 & 128.55 & 24.97\\ -37.78 & -74.16 & 111.93\\ 111.96 & -93.75 & -18.21 \end{array}\right]\left[\begin{array}{c} R\\ G\\ B \end{array}\right]+\left[\begin{array}{c} 16\\ 128\\ 128 \end{array}\right]. \end{array}$$

HSV color system models the human visual system to perceive the luminance, hue, and saturation better than the RGB color components. HSV can handle noise better than L ^∗^ a ^∗^ b ∗ and RGB color spaces. HSV color space has wide applications in medical images. In this work, we only consider the luminance image (V) of the texture image from the HSV color system, and the equations are shown below.

RGB to HSV conversion is explained here [44, 45]:

(4) $$\begin{array}{c} \text{Hue}=\left\{\begin{array}{cc} 60*\left(\frac{G-B}{\max\left(R,G,B\right)-\min\left(R,G,B\right)}\right) & R=\max\left(R,G,B\right)\\ 60*\left(\frac{2+\left(B|-|R\right)}{\max\left(R,G,B\right)-\min\left(R,G,B\right)}\right) & G=\max\left(R,G,B\right)\\ 60*\left(\frac{4+\left(R|-|G\right)}{\max\left(R,G,B\right)-\min\left(R,G,B\right)}\right) & B=\max\left(R,G,B\right) \end{array}\right.,\\ \begin{array}{c} \text{Saturation}=\frac{\max\left(R,G,B\right)-\min\left(R,G,B\right)}{\max\left(R,G,B\right)},\\ \text{Value}=\max\left(R,G,B\right). \end{array} \end{array}$$

The authors tested the proposed method with RGB, YCbCr, HSV, and L ^∗^ a ^∗^ b ^∗^ color space to investigate the efficiency of the luminance with YIQ color space.

#### 3.4.2. Generation of Deep-Luminance Feature Maps from Pretrained ResNet101 Model

Deep neural networks (DNN) are an important class of CNN that is widely used to capture the feature space and used for computer vision applications, including texture classification. The neural network learns these feature patterns automatically during the training process. DNN act as a feature extractor or a classifier for addressing pattern recognition problems. In DeepLumina, deep features are extracted from ResNet101 [46]. The pretrained models used for comparative analysis in this work are AlexNet [47], VGG19 [48], ResNet50 [46], ResNet101, Inceptionv3 [49], InceptionResNetv2 [50], DenseNet201 [51] and MobileNet [52]. AlexNet is the first popular CNN model proposed in 2012. AlexNet comprised of deep 60M parameters and used ReLU activation function and dropout to control the overfitting problem. VGG19 is a moderate CNN that has a large number of parameters. It is better than AlexNet in addressing the overfitting issue. ResNet50 and ResNet101 are popular and demanding pretrained models for texture analysis. The main idea behind solving the vanishing gradient problem in ResNet is the residual blocks that get repeated in the network. This network has several skip connections, and it learns and trains from the residual. In the inception model, the local, global, and skip connections features are captured from a 3 × 3 bock and 5 × 5 block, respectively. Large numbers of filters are applied, and convolution operations are performed. ResNet101 is a CNN with 101 layers depth. Each ResNet block is 3 layers deep. ResNet models have fewer filters and less complex. The number of trainable parameters in ResNet101 is 44,549,224.

The InceptionResNet model is a hybrid combination of the ResNet model and the Inception model where the residual inception blocks are designed. In DenseNet201, more dense blocks are present in the model, and this efficiently performs the error propagation. MobileNet has few parameters, and it is considered as a light-weighted model that applies the depth separable kernels. Gerihos et al. [53] investigated the prominence of the texture features captured from the pretrained CNN models trained on imageNet dataset. The convolution neural networks have the advantage of better classifying the textured images, even if the global structure and shape are not preserved in the image.

**Figure 6:**
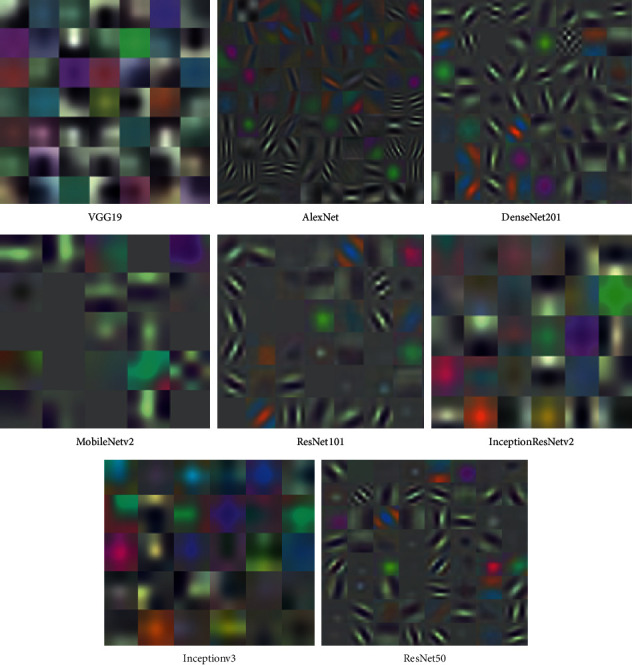
Visualization of the Deep Texture Feature Maps obtained from DeepLumina Framework.

DeepLumina constructs a feature map from the convolution layers. The deep network incorporates the RGB features and Y channel features, representing the color and the luminance information, respectively. This method produces a rich set of powerful and competent texture features with which the classification task becomes efficient. The convolutional layers capture the low and high level features from the images. The proposed method is computationally efficient since deep features are getting trained by an SVM classifier. The parameters for the convolution feature layer of different pretrained models in extracting the deep-luminance features are given in Tables 2 and 3. The parameters include the number of convolution layers used, input image size, kernel size, and feature layer. In glorot (xavier) initilization, weight initialization is based on the Guassian value with zero mean and variance based on the number of fan-in and fan-out hidden nodes. The feature maps obtained from the pretrained methods for the DeepLumina are visualized in Figure 6.

#### 3.4.3. Classification Using Support Vector Machine

Support Vector Machine (SVM) classifier has high generalization capability and constructs optimal hyperplane for categorizing the texture classes. It can be considered as a decision function, which is linear with a maximal margin between the vectors of texture classes. Krammer et al. [54] presented an approach for building multiclass support vector machines. SVM is formulated as an optimization problem. Different pixel intensities need to be classified in such a way so as to maximize the margin between the support vectors.

(5) $$\begin{array}{c} \begin{array}{c} \underset{\omega ,b,\psi }{\min}\frac{1}{2}w^{T}w+\lambda \sum_{i=1}^{n}\psi_{i}\\ y_{i}\left(w^{T}\phi \left(x_{i}\right)+b\right)\geq 1-\psi_{i} \end{array}, \end{array}$$

where *ψ*_i_ denotes the distance measure to the correct margin with *ψ*_*i*_ ≥ 0, *i*=1,…, *n*,where *λ* denotes a parameter for regularization,*w*^*T*^*w*=‖*w*^2^‖ denotes the normal vector,*ϕ*(*x*_*i*_) denotes the transformed new input space vector,*b* denotes a bias value,*y*_*i*_ denotes the i^th^ target value.

SVM proved to be a better classifier for texture classification since it can handle the high dimensional feature space found in texture patterns. Kim et al. [39] exploit the significance of the SVM classifier when applied for texture classification problems. SVM uses various kernel functions such as linear kernel, RBF kernel, and polynomial kernel. SVM is best suited for identifying texture patterns because of two significant reasons. First is the mapping of nonlinear texture space to a high dimensional space. Next is the optimal separating hyperplane construction. In this work, we use a multiclass support vector machine classifier with a fast linear solver to obtain features during training. For multiclass classification, the error-correcting output code (ECOC) [55] is utilized, which uses the outputs of binary learners to anticipate multitexture classes.

## 4. Results and Discussions

In this section, the experiments, result analysis, and comparison with other prior works have been discussed to analyze and evaluate the competence of DeepLumina.

### 4.1. Experimental Results

The DeepLumina model is tested on challenging datasets, namely DTD and FMD with ResNet101 CNN features using SVM classifier. The batch size for DeepLumina is selected as 27. The dataset is processed and it is split at random, with 80% of the images used during training and 20% used during testing. The loss function used for multiclass texture classification is categorical cross-entropy which captures the error rate in deep learning models. SVM performs classification using ECOC [55] model and uses sparse random as a coding parameter. In the proposed work, the FMD dataset obtained an accuracy of 90.15%, and the DTD dataset obtained an accuracy of 73.63%, which is a good improvement when compared with the existing state-of-the-art methods. The accuracy obtained for the DeepLumina for the datasets DTD and FMD is discussed in this section and presented in Tables 4 and 5. These tables illustrate the efficiency of the proposed method DeepLumina. The experiments are conducted in system configuration Intel(R) Core(TM) i7-8565U CPU @ 1.80 GHz, 8 GB RAM, NVIDIA GeForce MX150, Matlab.

We can infer from the experimental results that all the luminance-based color space conversions with RGB produce better results for texture classification. These results throw light into the significance of luminance features and also the YIQ color model. MobileNet model, a computationally efficient and light-weighted model, obtained an accuracy of 68.15% for YIQ. ResNet50 produced 73.01% for the YIQ model, and ResNet101 produced 73.63% in the YIQ model in the DTD dataset. In the DeepLumina framework, ResNet 50 and ResNet101 achieved competitive results in terms of accuracy when RGB-Luminance images and SVM are used. It is worth noticing that the color model YIQ produced the best results in ResNet pretrained models. In our work, we investigated the competence of the YIQ color model for texture classification. The combined RGB-Luminance information with deep networks demonstrates an excellent improvement in texture classification accuracy. MobileNet and ResNet50 model produces a better result of 87.3% and 89.7% with the L ^∗^ a ^∗^ b ∗ color space for the FMD dataset. In the DeepLumina framework, ResNet101 obtained excellent accuracy of 90.15% when RGB-Luminance images and SVM are used for the FMD dataset, which is very promising.

Texture data is high dimensional, and it provides a cue in visual inspection and object identification. From the experimental results, we can observe a 3% to 7% increase in the accuracy is obtained when we incorporated luminance information with RGB color space in both the datasets compared to the RGB color model. Pretrained CNN-FC models tested and compared show a steady improvement of accuracy, which is highly promising. FMD dataset obtained a high accuracy of 90.15%. We can infer that classification on the challenging texture datasets, namely. FMD and DTD, was efficient with the luminance features. DTD also gives a good result for ResNet50 and ResNet101 pretrained models. It provides a good result of about 73.63% because it can capture the fine and coarse texture details from the pretrained CNN architecture. We can also observe that the luminance from YIQ color model proved to be efficient in identifying the texture classes. Experiments demonstrate that the FMD dataset performs better in the ResNet101 model when used with color models L ^∗^ a ^∗^ b ^∗^, HSV, and YIQ color models. DTD dataset performs better in ResNet50 and ResNet101 models, both with YIQ color space.

### 4.2. Computation Time

The computation time obtained (in minutes) for the proposed DeepLumina method for the benchmark datasets DTD and FMD and the comparative analysis is given in Tables 6 and 7. MobileNet and AlexNet take significantly less computational time but obtains satisfactory accuracy improvement for the DTD dataset. InceptionResNetv2 and DenseNet201 models take high computation time (11 to 15 mins, respectively), but results are of comparable accuracy. ResNet50 and ResNet101 build computationally efficient models with good accuracy for the DTD dataset. The rest of the pretrained models compared for classifying the FMD dataset take nearly 0.5 to 3 minutes. InceptionResNetv2 model obtains good accuracy, but the execution time is comparable.

### 4.3. Comparison with CNN-FC Pretrained Models

**Figure 7:**
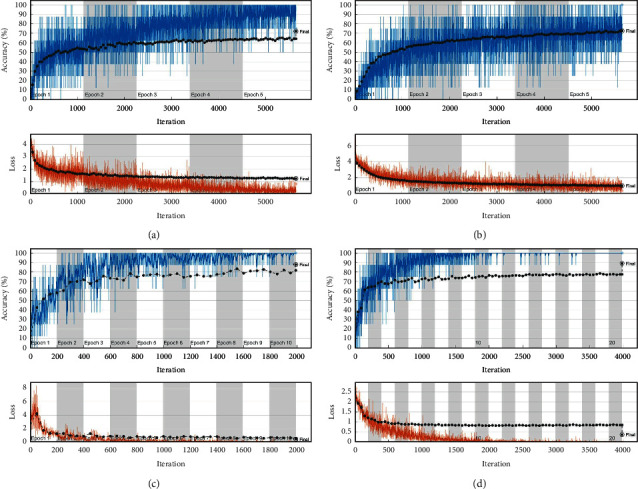
Accuracy-Loss curve for pretrained CNN-FC models with luminance information: Comparison.

In this section, the results obtained for the CNN-FC models are elaborated. The ResNet-FC CNN model with a fully connected layer is implemented for comparison with the proposed method. We performed an analysis with different end-to-end pretrained models for texture classification and analyzed the results. The experiments are conducted by training CNN pretrained networks using the RGB and RGB-Luminance information from the YIQ model and deduced inferences. Data Augmentation is performed with rotation, translation, and reflection on training data. We trained the CNN models on augmented data with epochs varying from 5 to 20. The batch size was fixed as 8. We performed tuning of hyperparameters of CNN with different values. The optimizer used is Stochastic Gradient Descent with Momentum (SGDM) and the learning rate used is 1e-05. We used ResNet50, ResNet101, AlexNet, VGG19, Inceptionv3, MobileNet, DenseNet201 for analysis. ResNet models have obtained an accuracy of 72.12% and 88% for DTD and FMD datasets. Also, VGG19 and Inceptionv3 performed better with accuracies 72.61% and 88.50% for DTD and FMD, respectively. From the results obtained, we inferred that processing texture images only in the RGB color system could not improve the performance. Luminance along with RGB information improved classification results. The results are summarized in Table 8. The computation time for the pretrained CNN-FC models ranges from 0.5 hours to 16 hours based on the hyperparameters specified for DTD and FMD datasets. This analysis shows that luminance has significance in classifying texture images, and using SVM as a classifier leads to computational efficiency. The experimental results of pretrained CNN-FC models with and without luminance in YIQ color space are depicted in Table 8 for DTD and FMD datasets. The accuracy-loss curve for the models with good accuracy is provided in Figure 7. From the experimental analysis, we inferred that DeepLumina had obtained better accuracy and computational efficiency than pretrained CNN-FC models.

### 4.4. Comparison with Prior Techniques

DeepLumina is compared with other state-of-the-art texture classification methods and results are highlighted in Tables 9 and 10. Cimpoi et al. [24] presented a custom fisher vector CNN (FV–CNN) and fully connected (FC–CNN) obtained an accuracy of 72.9% and 63.4% on the DTD dataset. This method is based on the VGG model, which extracts local features from the texture images. Simon et al. [18] discussed a deep framework that uses deep features from pretrained models and uses the support vector machine classifier to classify the textures in DTD and FMD datasets with 66.49% and 84.50% accuracy, respectively. Cimpoi [27] also proposed another method based on Improved Fisher Vector (IFV) and Deep Convolutional-network Activation Features (DeCAF), which obtained an accuracy of 66.7% and 65.5% for DTD and FMD dataset. Dai et al. [56] presented a Bilinear CNN model, FASON, that captured the second-order information within the features from the deep network and obtained an accuracy of 72.9% for the DTD dataset. Dai et al. incorporated a bilinear model with the first-order information fusion by gradient leaking, which captures the deep features and reduces the feature dimensions. Song et al. [25] showed the efficiency of the features obtained using CNN and designed a CNN for obtaining the features, performed dimensionality reduction. They achieved 83.2% accuracy on the FMD dataset. Cerezo et al. [57] analyzed the DTD dataset with ResNet50-FC and obtained 60.8% accuracy. Bell et al. developed a method based on Scale Invariant Feature Transform and Improved Fisher Vector (SIFT-IFV-fc7) and got 69.6 accuracies in the FMD dataset. The DeepLumina obtained a promising accuracy of 73.6% and 90.15% for DTD and FMD datasets from the ResNet101 Deep Features with the RGB and Luminance from YIQ Color Space classified by SVM.

## 5. Conclusion

Color Texture classification is a vital task performed in machine vision, especially in visual fine-grained recognition applications. The proposed work demonstrates the efficiency of introducing the luminance and RGB information in generating the deep features from the texture images. DeepLumina extracts a better texture feature map by incorporating the luminance features in the deep network. The experimental results show that DeepLumina takes minimal computation time. Experimental results also demonstrate the strong influence of luminance in the performance of the DeepLumina framework. In this work, we have tested using five-color models, namely RGB, YCbCr, YIQ, HSV, and L ^∗^ a ^∗^ b ^∗^ and used eight pretrained models, namely. MobileNet, ResNet50, ResNet101, DenseNet201, AlexNet, VGG16, Inceptionv3, and InceptionResNetv2. Using the DeepLumina method, higher accuracy of 73.63% and 90.15% are achieved in ResNet101 on the DTD and FMD datasets, respectively. The proposed method also throws light into the efficiency of YIQ color space in obtaining better texture classification accuracy.

## Figures and Tables

**Table 1:** DeepLumina framework.

DeepLumina -proposed method
Step 1. Resize the input RGB images and perform color space transformation to the YIQ model
Step 2. Estimation of luminance images (Y channel images) from YIQ color model
Step 3. Apply step 1 and step 2 images to the pretrained ResNet101 model
Step 4. Extraction of deep or learned feature map from the ResNet101
Step 5. Classification using a fast linear solver support vector machine

**Table 2:** CNN parameters used for feature map generation from the Pretrained Models

CNN parameters	ResNet50	AlexNet	Inceptionv3	DenseNet201
Convlayer name	conv1	conv1	conv2d_1	conv1 | conv
No.of layers	177	25	316	709
Input image size	[224,224,3]	[227,227,3]	[299,299,3]	[224,224,3]
Filter size	7 × 7	11 × 11	3 × 3	7 × 7
No of filters	64	96	32	64
Stride	[2,2]	[4,4]	[2,2]	[2,2]
Dilation factor	[1,1]	[1,1]	[1,1]	[1,1]
Padding size	[3,3,3,3]	[0,0,0,0]	[0,0,0,0]	[3,3,3,3]
Weight initialization	Glorot	Glorot	Glorot	Glorot
Feature layer	fc1000	fc8	Predictions	fc1000

**Table 3:** CNN parameters used for feature map generation from the Pretrained Models.

CNN parameters	ResNet101	MobileNet	VGG19	InceptionResNetv2
Convlayer name	conv1	conv_1	conv1_1	conv2d_1
No.of layers	347	155	47	825
Input image size	[224,224,3]	[224,224,3]	[224,224,3]	[299,299,3]
Filter size	7 × 7	3 × 3	3 × 3	3 × 3
No of filters	64	32	64	32
Stride	[2,2]	[2,2]	[1,1]	[2,2]
Dilation factor	[1,1]	[1,1]	[1,1]	[1,1]
Padding size	[3,3,3,3]	[0,1,0,1]	[1,1,1,1]	[0,0,0,0]
Weight initialization	Glorot	Glorot	Glorot	Glorot
Feature layer	fc1000	Logits	fc8	Predictions

**Table 4:** Accuracy obtained for DeepLumina on benchmark texture dataset DTD.

	RGB	Proposed method - DeepLumina			
**Pretrained models**	**RGB**	**RGB** **+** **Y**	**RGB** **+** **L**	**RGB** **+** **V**	**RGB** **+** **Y**
**ColorSpaces**	**RGB** 18	**YCbCr**	**L^∗^a^∗^b^∗^**	**HSV**	**YIQ**

MobileNet + SVM	61.37	66.64	67.31	66.14	**68.15**
ResNet50 + SVM	67.23	72.47	72.10	70.77	**73.01**
ResNet101 + SVM	66.92	72.50	72.31	72.43	**73.63**
DenseNet201 + SVM	65.12	70.66	70.64	68.35	**71.37**
AlexNet + SVM	46.35	49.75	49.42	48.71	**49.84**
VGG19 + SVM	55.78	58.60	58.86	58.73	**59.53**
Inceptionv3 + SVM	65.20	70.04	71.30	69.81	**71.18**
InceptionResNetv2 + SVM	65.94	70.78	71.29	70.85	**71.39**

Best values are shown in bold and they are obtained for the proposed Method DeepLumina for Luminance from the YIQ color model.

**Table 5:** Accuracy obtained for DeepLumina Method on benchmark texture dataset FMD.

	RGB	DeepLumina - proposed method			
**Pretrained models**	**RGB**	**RGB** **+** **Y**	**RGB** **+** **L**	**RGB** **+** **V**	**RGB** **+** **Y**
**ColorSpaces**	**RGB** [18]	**YCbCr**	**L** **^∗^** **a** **^∗^** **b** **^∗^**	**HSV**	**YIQ**

MobileNet + SVM	74.60	87.1	**87.30**	85.10	85.80
ResNet50 + SVM	81.60	88.45	**89.70**	87.85	88.80
ResNet101 + SVM	81.40	89.46	89.65	89.20	**90.15**
DenseNet201 + SVM	80.75	88.83	87.38	87.46	**89.17**
AlexNet + SVM	64.30	70.02	70.05	69.50	**71.50**
VGG19 + SVM	78.10	80.40	81.65	79.35	**81.68**
Inceptionv3 + SVM	76.60	88.70	88.15	87.55	**89.80**
InceptionResNetv2 + SVM	82.20	88.75	90.01	90.03	**90.05**

The best values obtained for DeepLumina on the FMD dataset are indicated in bold.

**Table 6:** Computation time(in mins) for the Proposed Method - DeepLumina on DTD dataset.

	RGB	DeepLumina: proposed method			
**Pretrained models**	**RGB**	**RGB** **+** **Y**	**RGB** **+** **L**	**RGB** **+** **V**	**RGB** **+** **Y**
**ColorSpaces**	**RGB** [18]	**YCbCr**	**L** **^∗^** **a** **^∗^** **b** **^∗^**	**HSV**	**YIQ**

MobileNet + SVM	1.76	2.98	2.96	3.06	3.04
ResNet50 + SVM	2.95	5.05	5.02	5.43	5.42
ResNet101 + SVM	4.91	8.8	8.78	8.71	9.29
DenseNet201 + SVM	6.28	11.64	11.39	11.66	11.51
AlexNet + SVM	1.89	2.18	2.33	2.20	2.41
VGG19 + SVM	5.17	9.16	10.01	10.19	9.51
Inceptionv3 + SVM	4.09	7.32	7.3	7.36	7.39
InceptionResNetv2 + SVM	7.95	15.69	15.33	15.59	15.53

**Table 7:** Computation time(in mins) for the Proposed Method, DeepLumina on FMD dataset.

	RGB	DeepLumina: proposed method			
**Pretrained models**	**RGB**	**RGB** **+** **Y**	**RGB** **+** **L**	**RGB** **+** **V**	**RGB** **+** **Y**
**ColorSpaces**	**RGB** [18]	**YCbCr**	**L** **^∗^** **a** **^∗^** **b** **^∗^**	**HSV**	**YIQ**

MobileNet + SVM	0.43	0.64	0.65	0.65	0.65
ResNet50 + SVM	0.62	0.97	0.98	0.97	0.98
ResNet101 + SVM	0.92	1.57	1.58	1.57	1.55
DenseNet201 + SVM	1.29	2.18	2.20	2.19	2.19
AlexNet + SVM	0.95	1.37	2.56	1.12	1.1
VGG19 + SVM	1.03	2.18	2.20	2.19	2.19
Inceptionv3 + SVM	0.87	1.47	1.46	1.46	1.48
InceptionResNetv2 + SVM	1.81	3.04	3.02	3.06	3.06

**Table 8:** Comparison with CNN-FC models with and without luminance for DTD and FMD Datasets.

Dataset	DTD		FMD	
**Pretrained**	**RGB**	**RGB** **+** **Y**	**RGB**	**RGB** **+** **Y**
**CNN-FC models**	**RGB**	**Luminance**		**Luminance**

MobileNet	59.49	65.50	68.50	85.00
ResNet50	63.20	**72.12**	75.21	**88.00**
ResNet101	64.10	70.50	75.56	87.50
DenseNet201	60.50	68.50	70.50	80.25
AlexNet	61.50	67.11	65.20	81.25
VGG19	64.20	**72.61**	77.50	84.00
Inceptionv3	62.50	69.50	78.50	**88.50**

The best accuracy obtained for CNN-FC models with and without luminance for DTD and FMD datasets is indicated in bold.

**Table 9:** Comparative Analysis for the DTD dataset.

Authors	Method	Accuracy
Cimpoi et al. [24]	FC-CNN	63.4 ± 0.9
Cimpoi et al. [24]	FV-CNN	72.9 ± 0.8
Simon et al. [18]	Deep features + SVM	66.49
Cimpoi et al. [27]	IFV + DeCAF	66.7
Dai et al. [56]	FASON (conv5)	72.3 ± 0.6
Dai et al. [56]	FASON (conv4 + conv5)	72.9 ± 0.7
Cerezo et al. [57]	ResNet50-FC	60.8
**DeepLumina (Proposed)**	Deep Features (ResNet101) + luminance + SVM	**73.63** ± **0.5**

**Table 10:** Comparative Analysis for the FMD dataset.

Authors	Method	Accuracy
Song et al. [25]	FC-CNN	78.1 ± 1.6
Song et al. [25]	FV-CNN	80.2 ± 1.8
Song et al. [25]	FC-CNN + FV-CNN	83.2 ± 1.6
Simon et al. [18]	Deep features + SVM	84.50
Cimpoi et al. [27]	IFV + DeCAF	65.5
Bell et al. [58]	SIFT-IFV + fc7	69.6 ± 0.3
**DeepLumina (proposed)**	Deep Features (ResNet101) + luminance + SVM	**90.15** ± **1.2**

The best accuracy value is indicated in bold.

## Data Availability

The data used to support the findings of this paper are publicly available benchmark datasets DTD and FMD, which can be found in the websites https://www.robots.ox.ac.uk/~vgg/data/dtd/ and https://people.csail.mit.edu/celiu/CVPR2010/FMD/
